# Back to Nature: a revival of natural strain improvement methodologies

**DOI:** 10.1111/1751-7915.12232

**Published:** 2014-12-09

**Authors:** Oscar P Kuipers

**Affiliations:** Molecular Genetics, University of GroningenNijenborgh 7, 9747 AG, Groningen, the Netherlands

Modern life sciences, biotechnology and synthetic biology are heavily dependent on state-of-the-art genetic engineering. This has led to great advances in biomedical and biotechnological applications, such as the identification of novel drug targets, the production of pharmaceuticals by use of novel engineered pathways, high-yield production of enzymes and, last but not least, the production of biofuels.

However, in some other large application areas, in particular food and environmental applications, the use of genetically engineered microbes, is far behind, or even non-existent (Pedersen *et al*., 2005; Derkx *et al*., 2014). Especially in Europe, but increasingly also in other parts of the world, there is still a solid opposition against the use of living engineered microbes in fermented foods, such as cheese, beer or sausage, or for their release into in the environment, e.g. to clean up pollution or enhance crop yields.

Though their scope and impact are virtually limitless, genetically modified organisms (GMOs) have faced societal resistance ever since their first mention. The main objections against the use of genetically modified microorganisms, commonly put forward by opponents of gene technology, are their presumed uncontrolled use, ‘unnatural'-ness, their likely spreading in the environment, possible negative effects on biodiversity, possible toxicity of new variants, supposed lack of good risk assessment protocols and negative effects on the economy of developing countries in view of intellectual property rights held mainly by multinationals. Genetically modified organisms are characterized by three main features: (i) the use of *in vitro* cloning of DNA with the use of restriction enzymes, ligases, PCR etc., (ii) the use of laboratory transformation protocols like electroporation, and (iii) the use of specific selection markers, frequently antibiotic resistance genes. This technology has proven to be very specific, making the risk of unwanted modifications very low. Moreover, current genomic technologies like re-sequencing of strains by next generation sequencing (NGS) can identify any undesired event. Taken together the vast majority of scientists do agree that safety offered by GMO is by far larger than that of classically modified organisms (e.g. UV, ethane methyl sulfonate-treated microbes) because these latter approaches give rise to large numbers of mutations or re-arrangements in the genome that have nothing to do with the desired phenotype. Despite this notion, many consumers and consumer organizations are reluctant to accept this and persist in expressing their (unfounded) fears. This debate has been going on for the last 30 years, without a good perspective for a change in opinion (Pedersen *et al*., 2005; Derkx *et al*., 2014). This might be the reason for the clear reluctance of any food company in Europe to include GMOs in their products.

However, the wish still exists to produce healthier, better tasting, longer lasting, less processed, and attractive looking and smelling foods. Is there a way to circumvent the problems stated above?

I think there is.

More than half a century ago, before the onset of genetic engineering, horizontal gene transfer was already known to occur in nature, via mechanisms such as conjugation, phage transduction and competence (McKay *et al*., 1973; Derkx *et al*., 2014). Thus, metal ion resistance or antibiotic resistance were found to occur regularly and could be transferred quite easily in nature. Subsequently, these mechanisms were studied in the following years, of course also by making use of genetic engineering (Barlow, 2009; Wozniak and Waldor, 2010; van Reenen and Dicks, 2011; Domingues *et al*., 2012; Baugher *et al*., 2014; Nielsen *et al*., 2014). It has become clear that horizontal gene transfer is an essential part of evolution and life, and that nature has developed many ways of transfer of useful genetic elements.

With the advanced understanding of these processes, combined with the potential of millions of microbial strains in nature, current powerful technologies of re-sequencing by powerful, cheap and fast NGS, the use of biomolecular markers such as GFP, experimental evolution approaches and use of the aforementioned natural transfer methods, will now enable the screening of large numbers of environmental strains for suitable plasmids, conjugative transposons, phages and other transfer vehicles m (McKay *et al*., 1973; van Reenen and Dicks, 2011; Domingues *et al*., 2012). These will be identified by genomics and by laboratory experiments to assess their suitability to function in natural horizontal gene transfer of desired elements (e.g. EPS gene cluster, adhesion genes, acid resistance genes, metabolic genes, flavour-promoting genes). I think this endeavour will not set us back 50 years, but instead will give us new chances to understand evolution and natural gene transfer, as well as to provide us the tools for the transfer of desirable traits from a donor organism to a recipient, which could be a strain used in real food or environmental applications (Pedersen *et al*., 2005; Ammann *et al*., 2008; Derkx *et al*., 2014). Of course, there are still several major challenges and problems to overcome. In the first place the selection method for the desired trait. This is straightforward if clear phenotypes are looked for, but more difficult when only small enzymatic changes are expected. Novel analytical techniques need to be developed to screen for live recipients having the desired trait. Second, the size of the cargo being able to be transferred is of importance, since desired properties might be multifactorial and residing on relatively large DNA fragments. Third, the host range to which traits can be transferred from one species to the other is crucial. If only one specific recipient can be used, this will greatly limit applicability in industry. Fourth, the stability of the genetic element transferred will determine whether or not the strain can and will be used in an industrial process. Considering these major challenges ahead, I believe that the road towards using natural horizontal gene transfer methods will be extremely rewarding. It will lead to a better understanding of the mechanisms of horizontal gene transfer, to identification and characterization of novel mechanisms as well as to practical solutions for obtaining improved microorganisms for use in production facilities and environmental applications. It remains to be seen how regulatory offices will respond to the technology for natural strain improvement, but dedicated risk assessment procedures based on re-sequencing of strains obtained, as well as thorough characterization of the new phenotype, should help to guarantee their safety. A detailed dossier of the strain should be provided, as is already common in many areas of industrial application of novel compounds or technologies. Although not everybody agrees, Rousseau (1712–1778) had good foresight when he used his crystal ball and stated his famous: Back to Nature.

## Conflict of interest

None declared.
